# Vascular and Perivascular Role in the Regulation of Angiogenesis: Impact on Arteriovenous Fistula Maturation

**DOI:** 10.26502/aimr.0185

**Published:** 2024-11-27

**Authors:** Fanya Xia, Vikrant Rai, Devendra K. Agrawal

**Affiliations:** 1 Department of Translational Research, College of the Osteopathic Medicine of the Pacific Western University of Health Sciences, Pomona, California 91766 USA

**Keywords:** Angiogenesis, Anti-angiogenic factors, Arteriovenous fistula, Extracellular matrix, Maturation failure, Pro-angiogenic factors, Vessel remodeling

## Abstract

Arteriovenous fistula (AVF) is a surgical connection between an artery and a vein created in patients with end-stage renal disease needing dialysis. A major concern with AVF is maturation failure which results, while creating a new AVF, a troublesome process for the patients. Thus, maturation of AVF is important which is achieved by outflow tract outward remodeling. However, vessel stenosis, hypoxia, endothelial dysfunction, and thrombosis contribute to AVF failure. Vascular stenosis and thrombosis after intimal injury due to intimal hyperplasia followed by plaque formation are major factors contributing to AVF maturation failure. Angiogenesis during plaque formation is important and plays a critical role but is also involved in vessel stenosis if uncontrolled. This suggests the dual role of angiogenesis and its effects on AVF maturation. Thus, it is critical to understand the factors regulating neoangiogenesis after the creation of AVF. Not only the angiogenesis in the plaque area but also in the adjoining tissues including muscles due to injury and the factors released by the perivascular structure may influence the angiogenesis and AVF maturation process. This review article comprehensively and critically discusses the role of neoangiogenesis in AVF maturation and the role of various factors regulating angiogenesis (pro- and anti-angiogenic factors) with their potential role in AVF maturation.

## 1. Introduction

An arteriovenous fistula (AVF) is an abnormal connection between an artery and a vein, created through a surgical process. AVFs are lifelines of end-stage renal disease (ESRD) patients receiving hemodialysis. The artificial connection of the artery and vein creates a more permanent access site for hemodialysis treatments since accessing the artery every time is not ideal. Before the AVF is created and matured, patients receive hemodialysis through a central venous catheter, which goes directly into a large vein. However, central venous catheters are associated with high rates of infection and sepsis with long-term use. After anastomosis, or surgical connection, is made between the adjacent vein and artery, the AVF must be matured, and the vein needs to adapt to the high pressure from the artery. The adventitia or the externa layer of the vein will eventually thicken to withstand pressure, the outward remodeling of the outflow vein, and then can be used for hemodialysis access [1,2]. While there is no official consensus, the National Kidney Foundation-Kidney Dialysis Outcomes Quality Initiative (NKF KDOQI) defines the maturation of an AVF in humans by the following criteria: fistula blood flow of 600 mL/min, vein diameter of 6 mm, and depth of 2 mm below the skin [3]. AVF maturation is regulated by various factors including inflammation, perivascular cuffing, transcription factors, and microRNAs [4-9].

A major concern with AVFs is the high rate of AVF maturation failure. This is often caused by stenosis (narrowing of the vessel lumen) or thrombosis (blood clot). Stenosis (which accounts for about 60% of AVF maturation failure) is characterized by neointimal hyperplasia, in which the intima layer of the vessel thickens and therefore decreases the lumen size, preventing adequate flow of the blood through the narrowed lumen. The process in which stenosis occurs is a positive feedback loop, beginning from vessel injury during the AVF procedure. Inflammation occurs after surgery, leading to intimal hyperplasia. Narrowed lumen diameter from remodeling causes blood velocity and pressure to increase. This increased flow has the potential to cause shear stress and injury on the arterial wall, leading to more inflammation, inward remodeling, and further lumen narrowing (positive feedback loop). Though this process is necessary to maintain adequate pressure and blood flow in the artery, if it goes unchecked it can cause stenosis and AVF maturation failure [5-10]. Thrombosis occurs from plaque buildup due to injury from shear stress and will eventually form a clot that breaks free and travels through the bloodstream. To understand the process behind thrombosis in AVF, it is important to understand Virchow’s Triad. Virchow’s Triad consists of three factors namely endothelial/vessel wall injury, hypercoagulability, and blood flow stasis predisposing to blood clot formation. After AVF surgery, plaque can build up due to endothelial injury to the artery. Due to increased oxygen demand in the developing angiogenesis in the plaque increases enlarging the plaque size, however, increased angiogenesis with time may lead to intraplaque hemorrhage resulting in plaque rupture [11]. Intimal hyperplasia and increasing plaque size due to intimal injury during AVF creation may result in early thrombosis and vessel stenosis contributing to AVF maturation failure [12]. Thus, neoangiogenesis may, in part, contribute to increasing plaque size and thrombosis.

Inflammation occurring after the surgery also signals an influx of immune cells to the area, furthering the inflammation. Chronic inflammation of the artery adventitia places the patient in a hypercoagulable state, in addition to other processes in ESRD patients. The combined effect of these factors leads to the buildup of atherosclerotic plaque. When the plaque builds to a certain point and breaks off, the clot is called thrombosis. These clots cause AVF failure when they are lodged in the artery space, preventing blood flow, and causing blood stasis. Plaque rupture may also be due to neovascularization within the plaque due to its increased vulnerability by enabling intraplaque hemorrhage, increased immune cells and inflammation, and increased extravasation of RBCs in the center of the plaque [5-7,10,13].

The role of inflammation in AVF maturation failure is well documented, however, the role of perivascular cuffing, transcription factors, microRNAs, and neoangiogenesis is not well understood. To understand the role of neoangiogenesis in AVF maturation failure, it is imperative to discuss the role of neoangiogenesis in vessel stenosis and thrombosis and the factors regulating neoangiogenesis. This review is a comprehensive discussion on the role of neoangiogenesis in vessel stenosis and thrombosis, their role in AVF maturation failure, angiogenic and anti-angiogenic factors, followed by novel factors which may regulate angiogenesis after AVF creation and affect AVF maturation. Briefly, we have also discussed the role of arteriogenesis and vasa vasorum in increasing blood supply in the presence of ischemia and their potential role in AVF maturation failure.

## 2. Neoangiogenesis, stenosis, and thrombosis

Neoangiogenesis is the formation of new blood vessels from a network of pre-existing vasculatures. It involves many processes like migration, proliferation, and differentiation of endothelial cells [14]. After AVF surgery, the body triggers an immune response due to endothelial injury. The injury triggers the release of local innate immune cells (neutrophils, macrophages, and dendritic cells (DCs)) and secretes damage-associated molecular patterns (DAMPs) like high mobility group box 1 (HMGB1), alarmins, and receptors for advanced glycation end products (RAGE). DAMPs further trigger the immune system and recruit more immune cells to the area through a cascade of inflammatory response [9]. In the artery, intimal injury from shear stress or AVF procedure is the primary driving force for remodeling. Shear stress is also capable of inducing atherosclerotic lesions and plaque formation. When pressures are high and injury is introduced to the artery, it can lead to stretching of the arterial walls and induce intimal hyperplasia [15]. Intimal hyperplasia can cause the vessel to become stenosed and its components including endothelial dysfunction and VSMC proliferation, migration, and phenotypic switch play a critical role in the maturation failure of AVF [16,17]. Increased intimal thickening, continued decrease in blood flow and hypoxia at the arterial site trigger the body to generate new vessels (neoangiogenesis) as an adaptive response to maintain adequate blood supply [9]. Neoangiogenesis is necessary to stimulate intimal thickening but not necessary to initiate intimal hyperplasia [18]. This is the physiological way to restore oxygen, nutrients, functions like macrophage infiltration, vessel wall thickening, lipid deposition, inflammation, and atherosclerotic lesion progression [19] to the hypoxic area. Furthermore, stretching of the arterial wall stimulates the proliferation of arterial smooth muscle cells (SMCs) [15].

SMCs are a key component in the formation and progression of atherosclerotic plaques. SMCs also contribute to the formation of fibrous cap, which is made up of collagen and elastin. This fibrous cap gives plaque its structure and when thick, it can prevent rupture. However, as atherosclerotic plaque enters more advanced stages, SMCs can become dysfunctional or undergo apoptosis. Less SMCs result in a thin fibrous plaque, which is a key characteristic of unstable plaques that can rupture and thrombose at any time [13]. Plaque rupture is facilitated by neoangiogenesis, decreased SMCs, and increased inflammation [19]. When plaques build up in the artery, the AVF fails either through stenosis or thrombosis (if ruptured). However, it should be noted that angiogenesis within the plaque is important for plaque growth in the early phases but is detrimental in the later phases and contributes to rupture [19]. Thus, it is important to understand the role of neoangiogenesis in plaque rupture, thrombosis, and AVF maturation/failure and the factors regulating this process.

## 3. Arteriogenesis and vasa vasorum

Atherosclerosis is characterized by changes to the inner vascular wall of medium and large sized arteries. The changes lead to the formation of atherosclerotic plaques, which may be the result of injury, inflammation, or a combination of both. There are four stages in the progression of atherosclerotic plaques. The first two stages (type I, II) consist of early lesions and contribute to intimal thickening, while the latter two stages (type III, IV) are advanced, calcified, and complicated [19-21]. In these areas of the artery, neoangiogenesis may occur from pre-existing vasa vasorum, to reroute blood flow from the stenosed artery.

Camaré and colleagues [19] note that the mechanism behind atherosclerotic lesions and intimal hyperplasia is angiogenesis from pre-existing vasa vasorum. In an ideal situation, when the artery experiences hypoxia, it triggers the body to release angiogenic factors like VEGF to begin angiogenesis. It is then balanced with downregulation when the body restores normoxia. Since neoangiogenesis plays an important role in the development of atherosclerotic plaques, it is important to investigate the mechanisms regulating neoangiogenesis in the early versus late stages of plaque formation.

Vasa vasorum is the microvasculature located in the outermost layer of blood vessels. These microvessels transport nutrients, oxygen, and other substances required to keep larger blood vessels healthy and functional. When blood flow is inadequate in the vessel lumen (due to intimal hyperplasia or atherosclerotic plaque), the vasa vasorum can extend from the adventitia to the media and even the intima layer to assist in the exchange of nutrients and waste [19]. During AVF surgery, the arterial and venous walls are damaged due to repeated clamping, lack of blood flow, and direct injury contributing to the damaged vasa vasorum. Injury and damage inflicted on the vasa vasorum have the potential to cause vasospasm, loss of distensibility, and decreased patency [22] in the artery, all of which can contribute to AVF failure. Thus, it is critical to minimize damage to the adventitia during the procedure. Post-surgery, vasa vasorum lining arterial and venous walls increased in size only, without evidence of new vessels being created [22]. This was probably due to the vessel receiving nutrients through the vasa vasorum, so there was no need for angiogenesis. The increase in the size of the vasa vasorum was not found to be associated with AVF maturation outcomes [22].

However, in regions of atherosclerotic plaques, the vessel density was higher than its healthy counterparts. Thus, the presence of increased angiogenesis in a certain area can indicate that arteriogenesis, the process of enlargement of existing collaterals to increase blood supply, may occur. Another possibility may be increased angiogenesis due to increased oxidative stress. Hypoxia caused by intimal hyperplasia triggers the growth of new blood vessels from the vasa vasorum in the adventitial layer. In more advanced stages, chronic inflammation continues to promote neoangiogenesis, but these new vessels become leaky and easily damaged due to oxidized lipids, oxidative stress, and other cytotoxic agents built up within the plaque. If this fragile vasculature is injured, hemorrhage can occur within the plaque, exacerbating oxidative stress to the plaque area [19,23,24]. This process can degrade the fibrin cap, increase plaque instability and risk of thrombosis, potentially resulting in AVF failure.

Arteriogenesis is the remodeling or enlargement of existing capillaries because of shear stress or obstructed flow in the artery. Arteriogenesis is important because it allows the artery to optimize blood flow without forming new branches of blood vessels. After AVF and occlusion of the artery via plaque formation or thrombosis, the body is driven to undergo arteriogenesis to form a more efficient flow. The ability of arteries to adapt to these changes suggests a capacity of the body to achieve maturation despite plaque buildup [25]. The process of arteriogenesis during the healing process is related to the restoration of the blood flow. When an artery is stenosed, arteriogenesis not only helps to restore perfusion by remodeling the artery but also improves artery function since it is now thicker and stronger. Arteriogenesis occurs in the vascular bed of the skeletal muscle during ischemia and increased arteriogenesis in the microcirculation (vessel diameters <35 μm) after ischemic ligations during arterial remodeling in a mouse model suggests that injury to the vessel may induce arteriogenesis in the perivascular structures [26]. Increased vasculature in the perivascular structures of AVF will result in increased immune cell recruitment and contribute to chronic inflammation via perivascular cuffing which ultimately may contribute to AVF maturation failure [6].

The extracellular matrix (ECM), made up of collagen and elastin fibers, plays an important role in the process of arteriogenesis. The ECM provides stability to the artery structure under hemodynamic stress; thus, ECM remodeling is necessary to achieve changes in the artery. In ECM remodeling, the lamella and internal elastic lamina (IEL) are digested by matrix metalloproteinases (MMPs), and then rebuilt to form a larger artery. When everything is rearranged, or remodeled, there is a possibility of disruption in elastin synthesis, and the elastin does not recover its original function [7,27]. This suggests that the remodeling process in arteriogenesis, which is also involved and necessary during outflow tract remodeling after AVF has the potential to result in AVF failure if ECM functions are lost [1,4]. This remodeling also assists in the prevention of ischemia to nearby tissues and helps in preventing AVF failure, since it is a solution for stenosed arteries. Since ECM remodeling plays a critical role in AVF maturation and arteriogenesis is critical for ECM remodeling [27-29], the role of arteriogenesis in AVF maturation or maturation failure will be of interest and should be investigated.

## 4. Regulation of neoangiogenesis

Neoangiogenesis is regulated by many pro-angiogenic and anti-angiogenic factors that work together to control the growth of new vessels. Angiogenesis is controlled by pro-angiogenic factors like vascular endothelial growth factor (VEGF) and fibroblast growth factors (FGFs). On the other hand, angiogenesis also needs to be regulated so that it does not go unchecked. This process is controlled by anti-angiogenic factors such as angiotensin 1 (Ang-1), pigment epithelium-derived factor (PEDF), angiotensins, endostatins, and thrombospondins [30]. Angiogenesis is also regulated by various proangiogenic and anti-angiogenic factors including hypoxia inducible factor 1 alpha (HIF-1α), Tie2 tyrosine kinase receptor, expression of VEGF receptors, angiopoietin-2 (Ang2), the density of pericytes, vascular smooth muscle cells (VSMCs), and endothelial cells (ECs), Sprouty2, Pigment epithelium-derived factor (PEDF; also known as SERPINF1), low-density lipoprotein receptor-related protein 6 (LRP6), thrombospondin 1 (TSP1), C-X-C motif chemokine ligand 10 (CXCL10) C-X-C chemokine receptor 3 (CXCR3), platelet derived growth factor receptor (PDGFR)-β, Heparin-binding EGF-like growth factor (HB-EGF), transforming growth factor (TGF)-β, epidermal growth factor receptor (EGFR), Neuropilin 1, Semaphorin-3A (SEMA3a), angiotensin II (Ang II), NG2, also known as chondroitin sulfate proteoglycan 4 (NG2; CSPG4), regulator of G-protein signaling 5 (RGS5), and others as reported using a mouse model of diabetes [30,31]. When the balance of these factors is disrupted, it can cause overgrowth of new vessels in the atherosclerotic plaques within the arterial walls, resulting in stenosis which will contribute to AVF failure. Thus, success in AVF maturation is dependent on the ability of these factors to balance each other out to create the optimal amount of angiogenesis.

ECM remodeling is a crucial event during arteriogenesis, and the laying down of vascular bed before neoangiogenesis is important because neovessels develop on a vascular bed that provides mechanical and nutritional support. Fibroblasts play a critical role in the secretion of ECM components (from myofibroblasts) and prepare a vascular bed for neoangiogenesis [32]. The newly secreted ECM is remodeled via MMPs and remodeled ECM helps in neoangiogenesis and arterial remodeling. MMP9 plays a crucial role in both angiogenesis and remodeling, which are essential for the maturation of AVFs. MMP9 degrades the ECM and facilitates endothelial cell migration [33]. Blood flow to the area of angiogenesis is also essential because its forces can regulate MMP9 activity and expression, contributing to the remodeling and maturation of AVFs. Blood flow critically regulates the vascular remodeling process by not only providing nutrients to the network itself, but also assists in the three processes in the vascular remodeling: pruning (selective segment removal), regression (complete loss of vessel), and fusion (merging) of the vessels. These processes are further governed by mechano-transduction, where physical forces like shear stress can activate important signaling pathways like VEGF. Then, these pathways initiate endothelial cell modulation, including processes like cell migrations, proliferation, and apoptosis. All of these are crucial to the formation of a functional and mature vascular network [34].

### 4.1. Factors Upregulating Neoangiogenesis

Upregulation refers to increase in vasculature which is regulated by various growth factors. VEGF is one of the best-known and well-studied growth factors in angiogenesis [35]. Fibroblast growth factor (FGF) is another well-known pro-angiogenic growth factor that is upregulated during the process of neoangiogenesis. In the early stages of plaque formation, factors known to increase neoangiogenesis include angiopoietins, inflammatory cytokines like tumor necrosis factor-alpha (TNF-α), and growth factors like VEGF or FGF. Angiopoietins (Ang-1 or Ang-2) are like growth factors involved in vascular remodeling. Ang-2 is a well-researched signaling molecule in angiogenesis and plays a crucial role in the early stage of angiogenesis. Ang-2 is significantly expressed in endothelial cells (ECs) lining the vessel lumen after exposure to angiogenic stimuli such as hypoxia and VEGF [35]. TNF-α, an inflammatory cytokine, is secreted by macrophages during angiogenesis when the inflammation or immune response is activated. At low levels, its role is to stimulate EC differentiation as well as the production/enhancement of pro-angiogenic factors like VEGF. VEGF plays a critical role in the upregulation of neoangiogenesis due to its ability to promote EC migration, proliferation, and formation of new blood vessels. This is important in the early stages of neoangiogenesis where development and remodeling of the blood vessel is necessary for AVF success. VEGF not only upregulates neoangiogenesis on its own but also signals the release of other factors to support vessel formation, making it a key target for both pro-angiogenic and anti-angiogenic therapies [35].

FGF is another growth factor that increases during blood vessel formation. The main two types, FGF-1 and FGF-2, find their respective receptors (FGFR-1 and FGFR-2) to promote EC migration and growth of neovasculature. FGF further plays a role by increasing VEGF production and assists in remodeling the ECM, which is essential in the maturation process of AVF. All these functions deem FGF essential in supporting and regulating neoangiogenesis in AVF. Interleukin (IL)-8 is another factor that regulates angiogenesis and is involved in ECM remodeling, regulation of inflammation, and atherosclerosis [36-38], however, its role in AVF maturation is unknown and should be investigated. TSP-2 is an ECM protein secreted from stromal fibroblasts, endothelial cells and immune cells which promotes angiogenesis and involves in ECM remodeling [39]. The role of TSP-2 in AVF maturation should be investigated to delineate its role after vascular injury. This notion is supported by the increased presence of immune cells in AVF tissues [8,9].

Angiopoietins bind to EC-specific Tie receptors, a group of receptor tyrosine kinases and play a role in vascular remodeling and angiogenesis. Ang-1 and Ang-2 bind the receptor Tie-2 stimulating downstream signaling while Ang-1 promotes angiogenesis and inhibits Ang-2, a negative regulator of angiogenesis [35]. Transforming Growth Factor-β (TGFβ) directly inhibits angiogenesis, however, it can promote angiogenesis via recruitment of immune cells promoting neoangiogenesis. Platelet-derived growth factors (PDGF)-BB has been approved by FDA to enhance wound healing based on the results of various clinical trials and this has been attributed to increased angiogenic response [40]. The role of various growth factors in promoting angiogenesis during wound healing has been investigated either in an animal model or in humans during clinical trials as discussed above. However, the role of growth factors secreted by the remodeling and perivascular structures on AVF maturation has not been investigated and warrants further research.

### 4.2. Factors Downregulating Neoangiogenesis

Downregulation refers to decrease in neovessel formation due to increased or decreased expression of genes or proteins. Angiostatin and endostatin are factors which inhibit angiogenesis. Angiostatin, derived from cancer-mediated proteolysis of plasminogen and pro-angiogenic plasmin, inhibits neoangiogenesis by attenuating VEGF expression and growth of neovessel formation. Angiostatin is produced by tumor cells [41]. Angiostatin is secreted by MMP12 and other MMPs and inhibits endothelial cell (EC) proliferation and promotes EC apoptosis [42]. Increased expression of MMP-2 and MMP-9 was found to be associated with AVF stenosis in rats [43], but inhibition of MMP-9 was associated with decreased vessel stenosis via decreased perivascular inflammation in mice [44]. Increased expression of MMP-9 was found to be associated with AVF maturation failure in a swine model [7]. This suggests that MMPs expression is increased after AVF creation and plays a critical role in AVF maturation, however, the expression and role of MMP-12 after AVF creation in fistula tissue and perivascular tissue has not been investigated. Delineating the expression and role of MMP-12 which produce angiostatin may also be important to investigate its role in angiogenesis which affects AVF maturation. Endostatin, a cleavage product of collagen XVIII, is a potent inhibitor of angiogenesis [45]. Endostatin attenuates endothelial cell proliferation and migration induced by VEGF [46]. The expression and the role of endostatin after vascular injury while creating AVF has not been investigated. Since endostatin attenuates angiogenesis, change in endostatin expression and its role in angiogenesis and AVF maturation warrant investigation.

TNF-α plays a role in the upregulation of angiogenesis, however, at high concentrations or during chronic inflammation, TNF-α can inhibit angiogenesis by initiating EC apoptosis and suppressing pro-angiogenic factor expression [35]. Thrombospondin 1 (TSP-1) is another anti-angiogenic factor that can directly inhibit key processes necessary for angiogenesis, such as endothelial cell migration, proliferation, and survival. It can also suppress the activation of MMPs, enzymes used in the degradation of ECM to allow endothelial cell invasion during angiogenesis [47]. Additionally, TSP-1 can bind and isolate pro-angiogenic factors like VEGF, FGF-2, and HGF, thus preventing their interaction with endothelial cell receptors and inhibiting angiogenesis [30]. TSP-1 also can induce apoptosis in endothelial cells, further contributing to its overall anti-angiogenic effects [48]. Investigating the role of TSP-1 in AVF maturation is important because TSP-1 expression increases after vascular injury in rat model [49] which will in turn attenuate angiogenesis and an early decrease in angiogenesis will contribute to AVF maturation failure.

Downregulation of neoangiogenesis carries equal importance as it provides the balance needed to prevent the overgrowth of microvasculature. Pathologic angiogenesis can be prevented by a few processes that occur to inhibit vascular growth in its later stages. The primary downregulator in neoangiogenesis is the ECM. The ECM mediates the entire process through a major basement membrane protein called type-IV collagen. Type-IV collagen is not only useful in elongating the new vessels when they begin sprouting but also in ensuring their continued growth into mature vessels. However, during the degradation/breakdown of type-IV collagen, it releases arrestin and canstatin, which halt the formation of new blood vessels [35]. The ECM, through the regulation of type-IV collagen and other degradation products, plays a critical role in the downregulation of neoangiogenesis. Keeping this balance is essential to prevent overgrowth of microvasculature after AVF surgery, which can lead to pathological conditions.

The role of pro- and anti-angiogenic factors after vascular injury or perivascular tissue injury in the proliferation, migration, and recruitment of endothelial cells and immune cells, as discussed above, suggest that these factors may play a role in AVF maturation or failure. However, their role in AVF maturation in the context of vascular as well as perivascular environment has not been investigated. Investigation of their role in AVF maturation is supported by the fact that these factors are critical in ECM remodeling, vessel remodeling, neoangiogenesis, and regulation of inflammation, the key determinants of AVF maturation. In addition to these factors with an existing role in angiogenesis, in the following section novel factors involved in tissue injury and angiogenesis are critically reviewed.

## 5. Novel Factors Regulating Neoangiogenesis

The factors discussed above upregulate or downregulate neoangiogenesis and are reported in the literature, mainly in the context of tumor biology, however, there is still a need to discover other factors that may play an important role in neoangiogenesis in various pathologies including AVF, diabetic foot ulcer, and peripheral arterial disease to improve the clinical outcomes. We researched the literature and the findings from our RNA sequencing (NCBI SRA submission # SUB11379495) to delineate other novel factors and found that the factors including MMP9, forkhead box protein C2 (FOXC2), transforming growth factor β-3 (TGFβ3), melanoma cell adhesion molecule (MCAM), SRY-Box Transcription Factor 7 (SOX7), parvin alpha (PARVA), adenosine deaminase (ADA), apolipoprotein A (APOE), uncoupling protein 3 (UCP3), fas apoptotic inhibitory molecule 2 (FAIM2), amyloid-beta precursor protein (APP), and myoblast determination protein 1 (MYOD1) may regulate neoangiogenesis.

MMP9 is known to be involved in neoangiogenesis through its role in thickening the arterial wall through intimal hyperplasia. An in vivo study using a human artery-SCID (severe combined immunodeficiency) chimera model [50] found that MMP9 promotes intramural neoangiogenesis and contributes to intimal hyperplasia. It does so by increasing levels of growth factors like VEGF, PDGF, and FGF, which promote upregulation of neoangiogenesis. The study also showed that high levels of MMP9 were correlated with pathologic angiogenesis and could lead to atherosclerotic plaque degradation and rupture, which is a potential cause of AVF failure. When inhibiting MMP9 using anti-MMP9 in a vasculitis mouse model, the study showed a decrease in neoangiogenesis and intimal hyperplasia. MMP9 also plays a role in ECM degradation and remodeling, which is important in keeping the balance to prevent pathologic angiogenesis and AVF failure. MMP9 has not been extensively studied but is shown to be a promising factor in regulating neoangiogenesis. An association of increased MMP9 expression with AVF maturation failure [7] suggest the notion of investigating the role of MMP9 in AVF maturation failure involving angiogenesis.

FOXC2 (Forkhead Box C2) is a transcription factor that is potentially involved in vascular formation during development [51] and may promote neoangiogenesis through its role in developing vascular and lymphatic systems, mostly studied in cancerous tumors. In a B16 mouse melanoma model, FOCX2 promotes vasculogenic mimicry in tumors, where the tumors begin to form pseudo-vessel structures that share similar properties to normal endothelial vessels. Vasculogenic mimicry is further amplified by FOXC2 during hypoxic states. Increased amounts of vessels in tumors are not ideal as these vessels can supply blood and other nutrients to the tumor to make it grow. Tumors that are affected by vasculogenic mimicry are also difficult to treat as they are resistant to anti-angiogenic therapies, so suppressing FOXC2 would allow for the cancers to be more receptive to treatments. Since FOXC2 contributes to vessel growth in tumors during hypoxia, it is worth studying whether it can replicate the effects of neoangiogenesis after the AVF procedure, where the surrounding tissue may show signs of hypoxia [52].

TGFβ3 is a family of TGFβ and induces downstream signaling that may be involved in regulating blood vessel formation and maintenance. It has been observed that cleft palate, a congenital anomaly in the craniofacial region, was associated with a lack of TGFβ3. A mouse cleft lip study [53] showed that lip tissues grown in TGFβ3 not only showed an increase in capillary vessels but were also able to fuse in just 2 days. This suggests that TGFβ3 plays a role in the regulation of neoangiogenesis, and its effects should be studied in the realm of AVF maturation.

MCAM (Melanoma Cell Adhesion Molecule), also known as CD146, is elevated when angiogenesis is deregulated. It has been found on endothelial cells, SMCs, and pericytes, all of which contribute to the formation of new blood vessels. In patients with diseases like cancer where angiogenesis is uncontrolled, MCAM is significantly elevated in the serum or interstitial fluid [54] of these patients. MCAM is involved in embryonic development, reproduction, wound healing, and tissue repair and modulates both physiological and pathological angiogenesis, suggesting that it plays roles in both upregulating and downregulating angiogenesis. This is another factor worth studying in relation to the inflammatory process and maturation of AVF.

SOX7 (SRY-Box Transcription Factor 7) is another transcription factor that has been found in an in vitro study [55] to upregulate in the human body when endothelial cells undergo hypoxic conditions. Hypoxia is a major driver in the angiogenic process, so it may play an important role for AVF patients. SOX7 activates pro-angiogenic genes like hypoxia-inducible factors (HIFs) in the initial stages of hypoxia, suggesting its role as an early regulator of angiogenesis. Furthermore, it was found in the study that expression levels differed within one hour of hypoxia, suggesting that SOX7 may be responsible for acute hypoxic responses, while unrestricted factors like VEGF are responsible for more chronic responses.

PARVA (Parvin Alpha) is a factor not commonly studied in relation to neo-angiogenesis, but it is known to be involved in cell adhesion and organization of cytoskeletons, which have potential effects on the formation of blood vessels. In an in vivo mouse model study [56], it was found that overexpression of PARVA promoted angiogenesis, tumor growth, and upregulation of VEGF-A expression. The silencing of endothelial-specific PARVA induced a reduction in VEGF-A and inhibition in tumor angiogenesis. The study results concluded that PARVA contributes to angiogenesis, but specifically in lung cancers. Future studies may utilize this information to study the effects of PARVA on angiogenesis outside of cancers.

ADA (Adenosine Deaminase) is an enzyme involved in breaking down adenosine in food as well as the replacement of nucleic acids. Its primary role resides in developing and maintaining the human immune system, but its role in neo-angiogenesis is not well studied. Further, deficiency in ADA promotes pulmonary angiogenesis through the CXCL1/CXCL2 pathway [57]. The ADA-deficient mice in the study exhibited significant angiogenesis in their trachea. This deficiency also caused elevated adenosine levels, which subsequently elevated CXCL1 levels. CXCL1 further promotes angiogenesis by interactions through the CXCL2 receptor, suggesting that ADA plays an important role in inducing pulmonary angiogenesis.

APOE (Apolipoprotein E) is a gene that metabolizes fats into lipoproteins. One version, APOE ε4, has been found to increase the risk of Alzheimer’s Disease and has been studied alongside VEGF [58]. VEGF signaling will increase angiogenesis. An in vivo study was done using DNA extracted from either peripheral blood monocytes (PBMCs) or brain tissue of elderly human patients collected at autopsy. The study showed that those who have the APOE ε4 gene were more likely to have Alzheimer’s disease. Previous literature showed that those with ε4 allele have “leaky” vessels in the brain, which poses a danger to newly forming microvasculature, which may be a cause of the onset of Alzheimer’s disease. Thus, angiogenesis may be risky in those with the APOE ε4 gene because the leaky vessels can lead to brain ischemia. Future studies on APOE ε4 should investigate its inhibition to strengthen blood vessels and improve neo-angiogenesis.

UCP3 (Uncoupling Protein 3) is an uncoupling protein that is mainly expressed in skeletal muscle. Its role is to regulate glucose and lipid metabolism in the body. UCP3 is upregulated in hypoxic conditions, which is a known driver for angiogenesis [59]. The in vitro study showed that upregulation of UCP3 contributed to hypoxia/reoxygenation (H/R) adaptation, while downregulation of UCP3 decreased H/R adaptation. Though there is no direct evidence of angiogenesis discussed in the study, the author suggests that the upregulation of UCP3 in renal cell carcinomas may contribute to cancer cell survival in hypoxic conditions, which in turn may promote angiogenesis within the cancerous tumor. However, the delineation of the specific mechanisms warrants further investigation.

FAIM2 (Fas Apoptotic Inhibitory Molecule 2) is a protein that regulates cell life and apoptosis. FAIM2 may play a role in angiogenesis through the regulation of calcium signaling pathways [60]. Bioinformatics analysis revealed that high expression of FAIM2 correlated with a significant increase in cell cycle turnover, suggesting faster formation of new cells. At the same time, the regulation of calcium was decreased. Decreased calcium signaling impacts the formation of new blood vessels. Thus, FAIM2 may also affect angiogenesis by decreasing it. However, future studies need to be done to better understand the relationship between FAIM2 and angiogenesis.

APP (Amyloid Precursor Protein) is a protein expressed in endothelial cells within the cerebral and peripheral arteries. APP is also highly expressed in the endothelium during fetal life, suggesting an important role for APP and/or its metabolites in early angiogenesis [61]. In this article, the authors observed through a mouse model that increased levels of VEGF-A, a major growth factor regulating angiogenesis, have been observed in mice with APP gene mutations. This suggests that APP may play a role in the regulation of VEGF and angiogenesis, particularly during early fetal developmental stages. While the exact mechanisms between APP and VEGF are unknown, there is potential to research this protein and its effects on blood vessel growth in future studies.

MYOD1 (Myogenic Differentiation 1), also known as MyoD, is a transcription factor that is involved in the activation, proliferation, and differentiation of satellite cells. Satellite cells eventually proliferate and develop into skeletal muscle cells to form muscles in the body. An in vivo study where myoblasts lacking the MYOD1 gene were transplanted into infarcted myocardium of mice [62] showed improvement in left ventricular function, likely due to increased angiogenesis in the heart. The article notes that the presence of MYOD1 seems to play a role in downregulating angiogenic genes. In high levels, MYOD1 suppressed the expression of angiogenic factors like SDF-1 and PIGF. In contrast, low levels of MYOD1 correlated to an increased level of pro-angiogenic factors (which can explain the improved left ventricular function in the mouse model). This relationship suggests that MYOD1 may play a role in interrupting the angiogenic process by putting a brake on important genes and factors that promote angiogenesis. Future studies of MYOD1 should investigate silencing or removing the MYOD1 factor to observe its effects on angiogenesis in the body.

## 6. Network analysis

**Figure 1:**
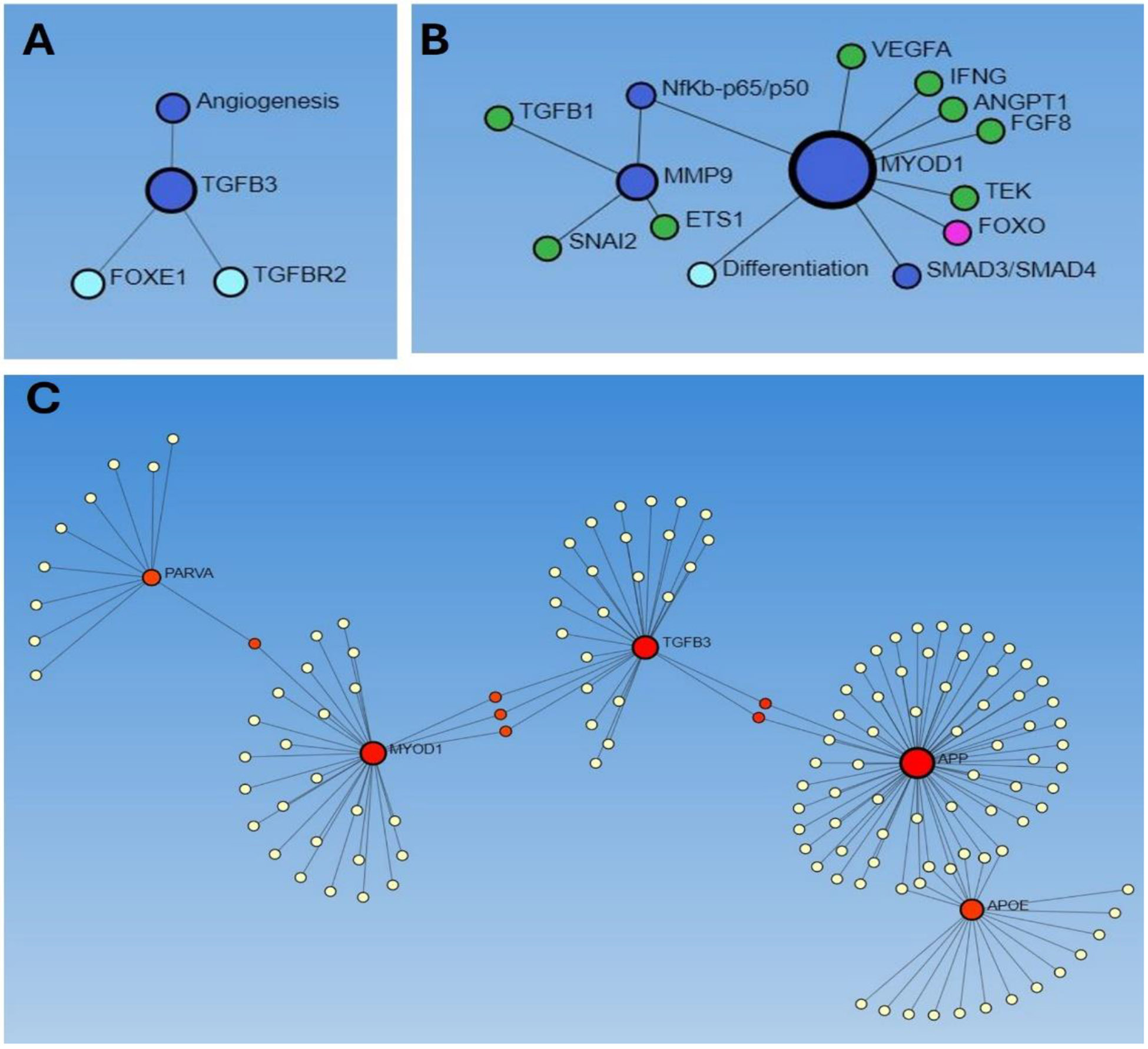
Network analysis using Networkanalyst.ca to predict the association between factors involved in neoangiogenesis. A: association of TGFβ3 with angiogenesis, B: association between MMP9 and MYOD1 and VEGF, and C: association between PARVA, MYOD1, TGFβ3, APP, and APOE.

The association of the factors involved in the process of angiogenesis, as discussed above, motivated us to do a network analysis to find the correlation of these factors with angiogenesis. We performed network analysis using Networkanalyst.ca with Search Tool for the Retrieval of Interacting Genes/Proteins (STRING) database and the results revealed an association of TGFβ3 with angiogenesis (Figure 1A), TGFβ1, VEGF, and MMP9 with MYOD1 (Figure 1B), and the association between PARVA, MYOD1, TGFβ3, APP, and APOE (Figure 1C). These results suggest that these factors may play a role in neoangiogenesis after AVF surgery. Further, ETS1 is known to regulate MMPs and collagens during atherosclerosis [20], both in turn as associated with ECM remodeling and the association of ETS1 with MMP9 suggest that MMP9 in association with ETS1 plays a role in angiogenesis. Further association between VEGF, MYOD1, IFNG, ANGPT, FGF8, and FOXO suggests their role in hypoxia-related angiogenesis, as discussed above. Next, an association between PARVA, MYOD1, TGFb3, APP, and APOE (Figure 1C) suggests their probable role in neoangiogenesis because each of these factors play a role in angiogenesis as discussed in the previous section.

**Figure 2:**
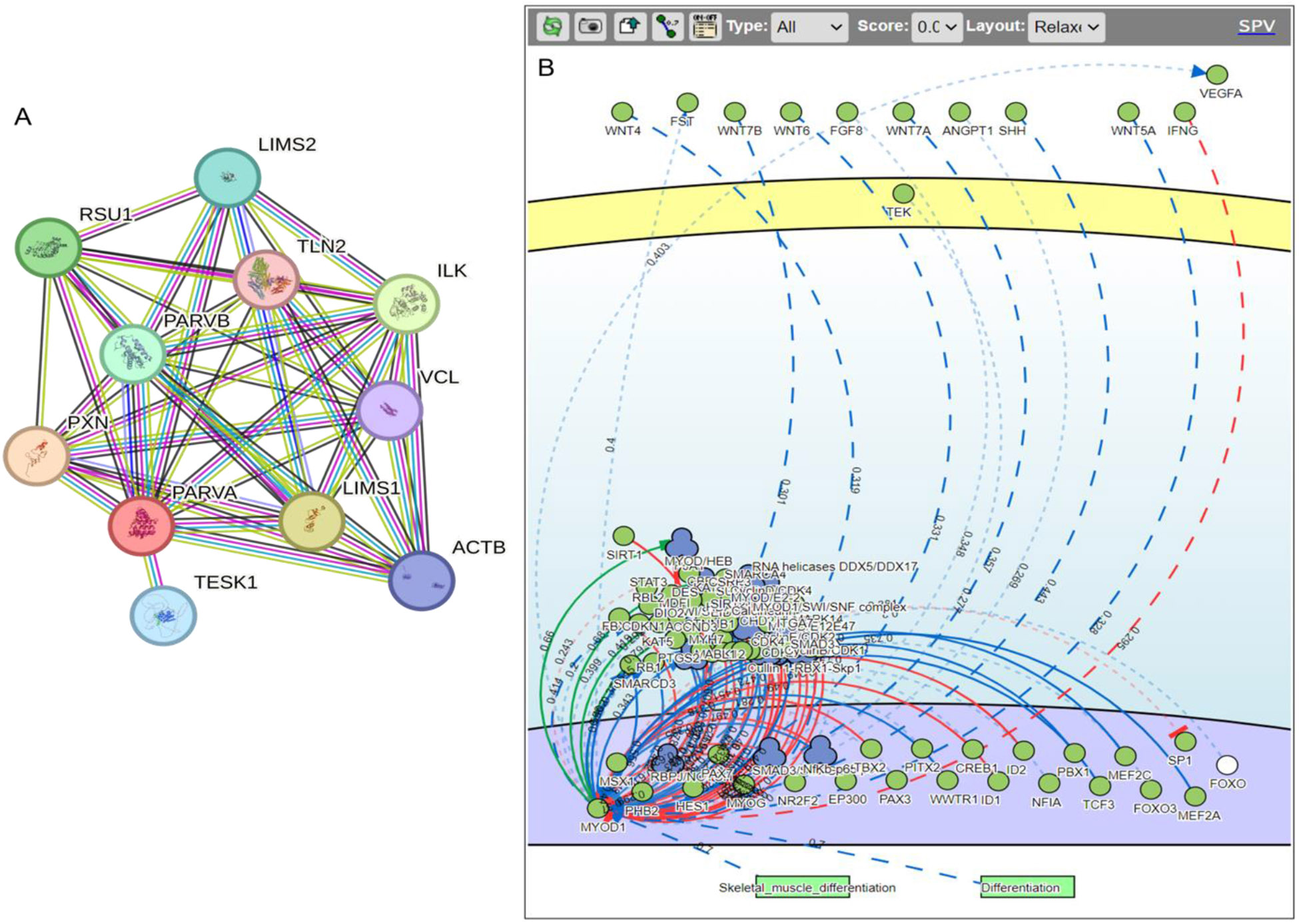
Search Tool for the Retrieval of Interacting Genes/Proteins (STRING) and SIGnaling network open resource (SIGNOR) network analysis of PARVA and MYOD1 to predict their role in angiogenesis.

Further network analysis using STRING suggest that PARVA (Figure 2A) plays a role in sprouting angiogenesis and is required for normal adhesion of vascular smooth muscle cells to endothelial cells during blood vessel development (https://string-db.org/cgi/network?taskId=bjOp0qsRGrD1&sessionId=bN7BwXkUK3LS). Inactivation of endothelial specific talin 1 (TLN1) is associated with increased angiogenesis [63], however, the role of TLN2 in angiogenesis is unknown. circRSU1 regulates retinal vascular endothelial cells and retinal microvascular function and promotes endothelial angiogenesis [64]. Vinculin (VCL), a mechanotransduction protein, controls endothelial cell junction dynamics and strengthens the endothelial barrier during angiogenesis [65]. Paxillin (PXN), a cellular adhesion molecule, promotes angiogenesis or arteriogenesis and is essential for VEGF-A-mediated angiogenesis in endothelial cells [66]. An association of PARVA with circRSU1, VCL, and PXN (Figure 2A) suggest that PARVA may play a role in angiogenesis during AVF maturation, however, further investigations are warranted.

SIGnaling network open resource (SIGNOR) analysis of PARVA revealed an association of PARVA with VEGFA, interferon gamma (IFNγ), fibroblast growth factor (FGF), ANGPT1, TEK, FOXOs, and others (Figure 2B) further suggest its role in controlling angiogenesis, however, this notion warrants investigation. IFNγ attenuates angiogenesis by decreasing EC proliferation in dextran sulfate sodium colitis in mice [67]. VEGF and FGF promote angiogenesis and their role have been discussed above. FGF-2 promotes angiogenesis via a SRSF1/SRSF3/SRPK1-dependent axis in ECs [68]. Angiopoietin-1 (ANGPT1) inhibits vascular inflammation, prevents endothelial cell death, has protective effects on vessels, and modulates angiogenic activity regulating the formation of the proper number and size of vessels, thus functioning as a “brake” to balance angiogenesis [69,70]. TEK tyrosine kinase (Tie2) is a receptor for ANGPT1 and regulates angiogenesis. Forkhead box O (FOXO) transcription factors play a regulatory role in angiogenesis and FOXO1 stimulates tip cell-enriched gene expression in endothelial cells promoting angiogenesis [71,72]. The interaction of PARVA with factors regulating angiogenesis suggest that PARVA may play a critical role in angiogenesis and AVF maturation warranting further investigations.

## 8. Conclusion

Angiogenesis is important for vessel remodeling and wound healing. Increased angiogenesis in a plaque may lead to vessel stenosis due to continued plaque growth. Decreased angiogenesis after intimal injury will result in altered ECM remodeling and wound repair. This suggests that a timely regulation of angiogenesis and the factors regulating neoangiogenesis, more importantly in the context of AVF maturation, should be investigated because ECM remodeling and vessel remodeling is critical for AVF maturation. Exploring the factors discussed in this article will not only the understanding on the role of angiogenesis in AVF maturation but also delineate therapeutic targets as well as serum biomarkers because expression of these proteins may change significantly after AVF creation.
